# Palonosetron and Ramosetron Compared for Effectiveness in Preventing Postoperative Nausea and Vomiting: A Systematic Review and Meta-Analysis

**DOI:** 10.1371/journal.pone.0168509

**Published:** 2016-12-19

**Authors:** EunJin Ahn, GeunJoo Choi, Hyun Kang, ChongWha Baek, YongHun Jung, YoungCheol Woo, SangSeok Lee, YeoGoo Chang

**Affiliations:** 1 Department of Anesthesiology and Pain Medicine, Inje University Seoul Paik Hospital, Seoul, Korea; 2 Department of Anesthesiology and Pain Medicine, Chung-Ang University College of Medicine, Seoul, Korea; 3 Department of Anesthesiology and Pain Medicine, Inje University Sanggye Paik Hospital, Seoul, Korea; 4 Department of General Surgery, Inje University Seoul Paik Hospital, Seoul, Korea; Jichi Medical University, JAPAN

## Abstract

Previous randomized controlled trials have reported conflicting findings on the superiority of palonosetron over ramosetron for preventing postoperative nausea and vomiting (PONV). Therefore, the present systematic review was registered in PROSPERO (CRD42016038120) and performed to compare the efficacy of perioperative administration of palonosetron to that of ramosetron for preventing PONV. We searched MEDLINE, EMBASE, and CENTRAL to identify all randomized controlled trials that compared the effectiveness of perioperative administration of palonosetron to that of ramosetron. The primary endpoints were defined as the incidence of postoperative nausea (PON), postoperative vomiting (POV), and PONV. A total of 695 patients were included in the final analysis. Subgroup analysis was performed through administration times which were divided into two phases: the early phase of surgery and the end of surgery. Combined analysis did not show differences between palonosetron and ramosetron in the overall incidence of PON, POV or PONV. Palonosetron was more effective than ramosetron, when the administration time for the 5-HT_3_ receptor antagonist was during the early phase of the operation. Otherwise, ramosetron was more effective than palonosetron, when the administration time was at the end of surgery. However, the quality of evidence for each outcome was low or very low and number of included studies was small, limiting our confidence in findings.

## Introduction

The etiology of postoperative nausea and vomiting (PONV) remains unclear, but patients still suffer from PONV with increasing healthcare costs and decreasing satisfaction [1,2]. The incidence of PONV when no antiemetics are administered is reported as high as 80%, and related to nearly all surgical procedures [3]. Therefore, numerous antiemetics, including antihistamines, anticholinergics, and dexamethasone, have been studied for the prevention and treatment of PONV. Among the available antiemetic drugs, palonosetron and ramosetron, which were both recently developed, are selective 5-hydroxytryptamine-3 receptor antagonists (5-HT_3_), which have a well-established role in the prophylaxis and treatment of PONV [4]. Palonosetron has a higher binding affinity to 5-HT_3_ receptors than do medications from the previous generation of 5-HT_3_ antagonists. Therefore, palonosetron has a significantly longer half-life (~40 hours) than dolasetron, granisetron, and ondansetron [5]. Ramosetron also shows a higher receptor affinity and longer duration of action than older agents in its class [6,7].

In numerous studies, researchers compared the efficacy of palonosetron to that of ramosetron in preventing PONV. However, the findings varied, and in several other studies, conflicting outcomes were reported. At the time of this writing, no systematic review or meta-analysis has been conducted to compare the effectiveness of palonosetron to that of ramosetron in the prevention of PONV. Therefore, we aimed to compare the effectiveness of palonosetron to that of ramosetron in preventing PONV.

## Methods

The present systematic review was registered in PROSPERO (CRD42016038120) and was conducted in accordance with the PRISMA statement guidelines [8] (S1 Checklist).

### Systematic search

We conducted a systematic review and meta-analysis of randomized controlled trials (RCTs) that compared the efficacy of palonosetron to that of ramosetron in preventing PONV. Studies in which single antiemetic was used were included. MEDLINE, EMBASE, the Cochrane Central Register of Controlled Trials (CENTRAL), Web of Science, Google Scholar and KoreaMed were searched for all relevant articles published by April 30, 2016 (inclusive). In addition, the reference lists of the full articles that were retrieved were searched manually. The search strategy, which combined free text and medical subject heading terms, is included in the S1 Appendix.

### Study selection

We determined the inclusion and exclusion criteria before the systematic search. Two authors (AEJ and CGJ) independently scanned the titles and abstracts of the reports identified via the search strategies previously described. If a report was determined to be eligible from the title or abstract, the full paper was retrieved. The full texts of potentially relevant studies chosen by at least one author were retrieved and evaluated. Articles that met the inclusion criteria were assessed separately by two authors (AEJ and KH), and any discrepancies were resolved through discussion. If no agreement could be reached, the dispute was resolved with the help of a third investigator (CYG).

### Inclusion and exclusion criteria

**Fig 1:**
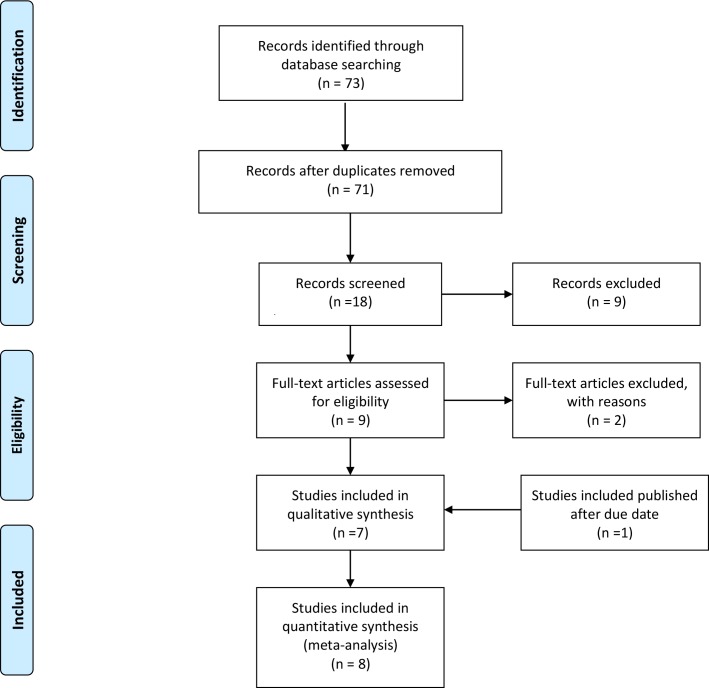
PRISMA flow diagram of the search for and the inclusion and exclusion of randomized controlled trials.

We included RCTs in which the efficacy of ramosetron and palonosetron on PONV prophylaxis were compared. We excluded data from abstracts, posters, case reports, comments or letters to the editor, reviews, and animal studies (Fig 1).

### Study outcomes

The primary endpoints were postoperative nausea (PON), postoperative vomiting (POV), and PONV. The occurrence of headaches and dizziness, were secondary outcomes in the systematic review.

Subgroup analysis was performed through administration times which were divided into two phases: the early phase of surgery and the end of surgery.

### Validity scoring

The quality of eligible studies was assessed independently by two members (BCW, JYH) of the review group by using the risk of bias tool from the Review Manager software program (version 5.3, The Cochrane Collaboration, Oxford, UK). We evaluated the quality of the study on the basis of the following seven potential sources of bias: random sequence generation, allocation concealment, blinding of the participants, blinding of outcome assessment, incomplete outcome data, selective reporting, and overall risk bias. The data were then cross-checked. The methodology of each trial was graded as ‘high,’ ‘low,’ or ‘unclear’ to reflect a high risk of bias, a low risk of bias, or uncertainty as to the risk of bias [9].

### Data extraction

Two authors independently extracted all interrelated data from the included studies and entered them into a spreadsheet (AEJ, SSL). The data were then cross checked. All discrepancies were resolved through discussion. If an agreement could not be reached, the dispute was resolved with the help of a third investigator (KH). The spreadsheet included the following items: (1) title; (2) authors; (3) name of journal; (4) publication year; (5) study design; (6) registration of clinical trial; (7) competing interests; (8) country; (9) risk of bias; (10) number of patients in study; (11) doses of palonosetron and ramosetron; (12) sex; (13) age; (14) weight of patients; (15) height of patients; (16) duration of anesthesia; (17) American Society of Anesthesiologists physical status; (18) inclusion criteria; (19) exclusion criteria; (20) type of surgery; (21) type of anesthesia; (22) induction agent; (23) maintenance agent; (24) use of nitrous oxide; (25) use of an opioid during the perioperative period; (26) timing of administration of the experimental drug (either palonosetron or ramosetron); (27) other drugs used during surgery; (28) timing of rescue antiemetics; (29) rescue analgesics; (30) definitions of nausea, vomiting, and retching; (31) number of cases of PON, POV, and PONV overall and during the early, late postoperative phases; and (32) the need for rescue antiemetics.

The data were extracted from tables or text initially. In the case of missing or incomplete data, we attempted to contact the study authors to obtain the relevant information.

### Statistical Analysis

The review and meta-analysis were conducted by using Review Manager. For dichotomous data, a pooled risk ratio (RR) and 95% confidence intervals (CIs) were calculated. If the 95% CI included a value of 1, we considered the difference to not be statistically significant. We calculated the mean difference (MD) for continuous data and also reported the 95% CI. If the 95% CI included a value of 0, we considered the difference to not be statistically significant.

We used the Chi-squared test and the I-squared test for heterogeneity. If the P value was <0.10 or the I^2^ value was >50%, we considered this indicative of significant heterogeneity. We selected a fixed effects model if the I^2^ value was <50%; otherwise, a random effects model was used. Because fewer than 10 studies showed substantial heterogeneity, t-test (Hartung-Knapp-Sidik-Jonkman method) were used instead of the Z-test in all random effects analyses to lower the error rate. A subgroup analysis was based on the timing of the assessment of PON, POV, and PONV and the time of the administration of the 5-HT_3_ antagonist.

We calculated the number needed to treat (NNT) with a 95% CI on the basis of the absolute risk reduction as an estimate of the overall clinical impact of the intervention [10].

### Evidence synthesis

The evidence grade was determined using the guidelines of the GRADE (Grading of Recommendations, Assessment, Development, and Evaluation) system which uses sequential assessment of the evidence quality that is followed by an assessment of the risk–benefit balance and a subsequent judgment on the strength of the recommendations [11]. The evidence grades are divided into the four categories as follows: (1) high indicates that further research is unlikely to alter confidence in the effect estimate; (2) moderate indicates that further research is likely to significantly alter confidence in the effect estimate and may change the estimate; (3) low indicates that further research is likely to significantly alter confidence in the effect estimate and to change the estimate; and (4) very low indicates that any effect estimate is uncertain. The evidence grade was lower or raised by measuring the uniformity of the estimated effects across studies and the extent to which the patients, interventions and outcome are similar to those of interest. As recommended by the GRADE working group, the lowest evidence quality for any of the outcomes was used to rate the overall evidence quality. The evidence quality was graded using the GRADE pro Version 3.6 software. The strengths of the recommendations were based on the quality of the evidence.

## Results

### Literature search and study characteristics

From searches of MEDLINE, EMBASE, CENTRAL, Web of Science, Google Scholar and KoreaMed 73 studies were initially evaluated. After excluding duplicates, 71 studies remained. Of these, 53 were excluded because they were considered irrelevant after their titles and abstracts were reviewed. Kappa value for selecting literatures between two reviewers are 0.794. Of the remaining 18 studies, seven were excluded because they were review articles and two were excluded because they were designed for chemotherapy-induced nausea and vomiting (Kappa = 0.896). The full texts of the remaining nine studies were reviewed in more detail; two more studies were excluded because one did not include palonosetron or ramosetron [12] and one was an abstract, not a published article [13]. An additional study, found through a Google search, met the inclusion criteria and was included in this meta-analysis [14]. Thus, eight studies with a total of 695 patients were included in the final systematic review and meta-analysis [14–21] (Fig 1).

The characteristics of the eight studies that met the inclusion criteria are summarized in Tables 1, 2 and 3. The postoperative period was divided into three phases: early (0–6 hours after surgery), late (24–48 hours after surgery) and overall phase. The overall phase was included to capture the maximum number of studies that contained PON, POV and PONV data with a variable data collection period, and was defined as the first period of data collection. Because several studies defined the early and late phases differently, we combined the various periods of data collection with early and late periods. For the early phase, one study included data collected 0–1 hours [16] postoperatively, one study included data collected 2–6 hours [21] postoperatively, and one study included data collected upon arrival at post-anesthesia care units (PACU)[17].

**Table 1:** Data Extracted from the Included Studies.

Source	Risk factors for PONV	ASA PS	Age	Duration of Anesthesia (min)	Type of Surgery		Type of Anesthesia
Chattopadhyay 2015 [15]	≥2 (female, non smoking)	Ⅰ-II	18–35	60.5[4.1]	elective cesarean delivery	Spinal anesthesia	
Kim 2013 [16]	≥3 (female, IV-PCA, non smoker)	Ⅰ-Ⅱ	20–65	169.39[87.6]	Laparoscopic surgery	General anesthesia	
Kim 2015 [17]	≥2 (female, IV-PCA)	not mentioned	not mentioned	146[44]	Gynecologic laparoscopic surgery	General anesthesia	
Lee 2015 [18]	≥1 (female)	Ⅰ-Ⅱ	not mentioned	128.1[47.5]	Laparoscopic hysterectomy	General anesthesia	
Park 2013 [19]	≥1 (IV-PCA)	Ⅰ-Ⅱ	≥ 20	143.4[53.8]	Gynecologic laparoscopic surgery	General anesthesia	
Roh 2014 [20]	≥1 (IV-PCA)	not mentioned	20–65	168[66]	Lumbar spinal surgery	General anesthesia	
Swaika 2011 [21]	≥1 (female)	Ⅰ-Ⅱ	18–70	56.1[8.0]	Laparoscopic Cholecystectomy	General anesthesia	
Yatoo 2016 [14]	≥0	Ⅰ-Ⅱ	18–65	42.6[9.4]	Elective laparoscopic surgery	General anesthesia	

ASA, American Society of Anesthesiology classification; IV-PCA, intravenous patient controlled analgesia; PONV, postoperative nausea and vomiting. Values of weight and height are mean [SD].

**Table 2:** Further Data Extracted from the Included Studies.

Source	Induction agent	Maintenance agent	Administration timing	Palonosetron/Ramosetron	Rescue analgesics
Chattopadhyay 2015 [15]	0.5% heavy bupivacaine	None	immediately after clamping of the fetal umbilical cord.	0.075mg/0.3mg	Diclofenac 75 mg, paracetamol 1 g
Kim 2013 [16]	2 mg/kg of propofol and 1 μg/kg of remifentanil infusion, 0.6mg/kg of rocuronium	Sevoflurane, remifentanil	just prior to induction of anesthesia.	0.075mg/0.3mg	ketorolac 30mg
Kim 2015 [17]	lidocaine 0.5mg/kg, propofol 2mg/kg, remifentanil infusion, rocuronium 0.6mg/kg	sevoflurane	10 min at the end of surgery	0.075mg/0.3mg	ketorolac 0.5 mg/kg and fentanyl 0.2 μg/kg
Lee 2015 [18]	propofol (target effect site concentration 2.5–3.5 μg/ml) and remifentanil (target effect site concentration 2.5–5.0 ng/ml), rocuronium 0.6mg/kg	sevoflurane	at the end of the surgery, prior to extubation	0.075mg/0.3mg	diclofenac 75 mg
Park 2013 [19]	propofol 2mg/kg, rocuronium 0.6mg	sevoflurane	immediately before the induction of anesthesia	0.075mg/0.3mg	IV-PCA
Roh 2014 [20]	1.5 to 2.5 mg/kg of propofol, 0.5 to 1.5 μ g/kg of Remifentanil, and 0.06 mg/kg of Rocuronium	1.5% to 2.5% of Sevoflurane, 0.1 to 0.3 μ g/kg/min of remifentanil	Ten minutes before the end of surgery	0.075mg/0.3mg	30 mg of ketorolac
Swaika 2011 [21]	thiopentone sodium 3 to 5 mg/kg, suxamethonium 1.5 mg/kg	sevoflurane (0.5–1%), nitrous oxide (60%), and atracurium (0.5 mg/kg)	just at the end of surgery before extubation	0.075mg/0.3mg	diclofenac 75 mg, butorphanol 2 mg
Yatoo 2016 [14]	propofol 2mg/kg, rocuronium 0.6mg	Halothane 0.5–1%, nitrous oxide 50%	five minutes before the induction	0.075mg/0.3mg	diclofenac 75 mg

IV-PCA, IV-patient controlled analgesia

**Table 3:** Further Data Extracted from the Included Studies.

Source	Number of patients	Sex (Male/Female)	Weight(kg)	Height(cm)	Rescue antiemetics	Data collection period	
Chattopadhyay 2015 [15]	109	0/109	58.8[7.2]	not reported	metoclopramide 10 mg		0-2/2-24/24-48h
Kim 2013 [16]	74	0/74	65[11.3]	164.5[4.9]	First choice, propofol 20mg, metoclopramide 10mg; Second choice ondansetron 4mg or/and dexamethasone 4mg		0-1/1-6/6-24/24/48h
Kim 2015 [17]	88	0/88	59[9]	158[5]	metoclopramide 10 mg		Arrival PACU/Discharge PACU/24h/48h/72h
Lee 2015 [18]	70	0/70	60.1[4.9]	155.3[3.1]	metoclopramide 10 mg		0-6h/6-24h/24-48h
Park 2013 [19]	100	0/100	61.8[8.5]	158.9[5.8]	metoclopramide 10 mg		0-6h/6-24h/24-48h
Roh 2014 [20]	196	107/89	not reported	not reported	metoclopramide 10 mg		PACU/0-6h/6-24h/24-48h/48-72h
Swaika 2011 [21]	58	0/90	52.8[6.9]	not reported	ondansetron 4 mg		0-2h/2-6h/6-24h
Yaoo 2016 [14]	60	31/29	65.4[4.8]	157.4[7.2]	metoclopramide 0.15 mg/kg		0-4h/4-12h/24-48h

PACU, post anesthesia care unit. Values of weight and height are mean [SD].

The 5-HT_3_ antagonists (palonosetron and ramosetron) were administrated during the early phase of the operation in four studies [14–16,19] and at the end of the surgery in four [17,18,20,21].

### Risk of bias

Seven studies mentioned the use of random sequence generation, and allocation concealment was used in five studies. In every study, outcome assessors were blinded and there were no incomplete data. In one study, outcome assessment was not blinded. The overall risks of bias are shown in Table 4.

**Table 4:** Risk of Bias in the Included Randomized Controlled trials.

Biases/ References	Random sequence generation	Allocation concealment	Blinding of outcome assessment	Incomplete outcome data	Selective reporting	Other bias	Overall risk of bias
Chattopadhyay 2015 [15]	low risk	low risk	low risk	unclear	unclear	low risk	unclear
Kim 2013 [16]	low risk	low risk	low risk	low risk	low risk	low risk	low risk
Kim 2015 [17]	low risk	low risk	low risk	unclear	low risk	low risk	unclear
Lee 2015 [18]	low risk	unclear	unclear	unclear	low risk	low risk	unclear
Park 2013 [19]	low risk	low risk	low risk	low risk	low risk	low risk	low risk
Roh 2014 [20]	low risk	low risk	low risk	unclear	low risk	low risk	unclear
Swaika 2011 [21]	low risk	unclear	low risk	unclear	unclear	low risk	unclear
Yatoo 2016 [14]	low risk	unclear	unclear	low risk	unclear	low risk	unclear

Method of estimating overall risk of bias: If all results or the above items were “low risk”, the overall risk of bias of the trial was deemed to be low risk of bias. If more than one of the above items were “unclear” or “high risk”, the overall risk of bias of the trial was deemed to be unclear risk of bias or high risk of bias, respectively. High risk indicates high risk of bias; low risk, low risk of bias; unclear risk, unclear risk of bias because of lack of detailed reports.

### PON (early, late and overall phases)

Six studies [14–16,18–20] compared the effectiveness of palonosetron to that of ramosetron in the prevention of early PON. Five studies [15,16,18–20] compared late PON, and six studies [14–16,18–20] compared the effectiveness of the drugs on overall PON. There were no significant differences between palonosetron and ramosetron in the incidence of early PON (RR 0.92; 95% CI, 0.54 to1.58; P_chi_^2^ = 0.06; I^2^ = 53.1%; Number needed to treat harm(NNTH) 240.8; 95% CI, NNTH 13.4 to ∞ to Number needed to treat benefit (NNTB) 15.2), late PON (RR 0.87; 95% CI, 0.48 to 1.57; P_chi_^2^ = 0.061; I^2^ = 55.5%; NNTB 57.3; 95% CI, NNTH 19.7 to ∞ to NNTB 11.7), or overall PON between palonosetron and ramosetron (RR 0.92; 95% CI, 0.54 to1.58; P_chi_^2^ = 0.06; I^2^ = 53.1%; NNTH 240.8; 95% CI, NNTH 13.4 to ∞ to NNTB 15.2).

### POV (early, late and overall phases)

**Fig 2:**
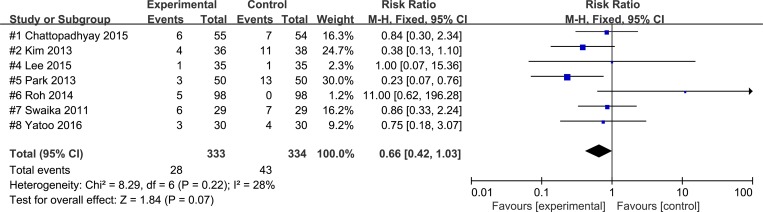
Forest plot for studies comparing the effect of palonosetron to that of ramosetron on overall POV. The figure depicts individual trials as filled squares with relative size of sample size and solid line as the 95% confidence interval of the difference. The diamond shape indicates the pooled estimate and uncertainty for the combined effect.

Palonosetron and ramosetron were compared in six studies [14,15,18–21] for their effectiveness on early, and in four studies for their effectiveness on late [15,18–20] and in seven studies for their effectiveness on overall POV [14–16,18–21]. The combined results did not reveal significance differences between the effectiveness of palonosetron and that of ramosetron in the incidence of early POV (RR 0.75; 95% CI, 0.46 to 1.23; P_chi_^2^ = 0.20; I^2^ = 31%; NNTB 36.6; 95% CI, NNTH 50.6 to ∞ to NNTB 13.4), late POV (RR 0.66; 95% CI, 0.39 to 1.14; P_chi_^2^ = 0.84; I^2^ = 0%; NNTB 26.1; 95% CI, NNTH 82.3 to ∞ to NNTB 11.3) or overall POV(RR 0.66; 95% CI 0.42 to 1.03; P_chi_^2^ = 0.22; I^2^ = 28%; NNTB 22.4; 95% CI, NNTH 495.8 to ∞ to NNTB 10.9). (Fig 2)

### PONV(early, late and overall phases)

Five studies [14,15,17,19,20] assessed the effectiveness of palonosetron and of ramosetron on early and four studies assessed the effectiveness of each drug on late PONV [15,17,19,20] and seven studies on overall PONV [14,15,17–21]. The combined results could not reveal the differences between the effectiveness of palonosetron and that of ramosetron on early PONV (RR 1.07; 95% CI, 0.60 to 1.92; P_chi_^2^ = 0.03; I^2^ = 63%; NNT 25.8; 95% CI, NNTH 8.7 to ∞ to NNTB 26.3), late PONV (RR 0.92; 95% CI 0.72 to 1.19; P_chi_^2^ = 0.14; I^2^ = 44.4%; NNTB 74.1; 95% CI, NNTH 14.4 to ∞ to NNTB 10.4) or overall PONV (RR 1.23; 95% CI 0.82 to 1.85; P_chi_^2^ = 0.034; I^2^ = 56.1%; NNTB 14.4; 95% CI, NNTH 7.2 to ∞ to NNTB 1957.8).

#### Headache

Five studies [15–17,19,20] compared palonosetron recipients to ramosetron recipients for the incidence of headaches. Analysis of the combined findings indicated no significant differences between the groups with respect to headaches (RR 1.06; 95% CI, 0.66 to 1.70; P_chi_^2^ = 0.984; I^2^ = 0.00%; NNTH 134.6; 95% CI, NNTH 17.2 to ∞ to NNTB 23.1).

#### Dizziness

The overall effects of palonosetron and ramosetron on the incidence of dizziness was assessed in five studies [15,16,17,19,20], but no significant differences were observed between the two groups (RR, 0.91; 95% CI, 0.55 to 1.49; P_chi_^2^ = 0.334; I^2^ = 12.48%; NNTB, 72.7; 95% CI, NNTH 27.7 to ∞ to NNTB 15.7).

### Subgroup analysis

#### The timing of antiemetics administration

**Fig 3:**
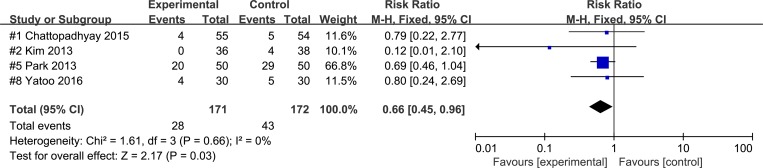
Forest plot for studies comparing the effect of palonosetron and to that of ramosetron on overall PON when the administration time was during the early phase of surgery. The figure depicts individual trials as filled squares with relative size of sample size and solid line as the 95% confidence interval of the difference. The diamond shape indicates the pooled estimate and uncertainty for the combined effect.

**Fig 4:**
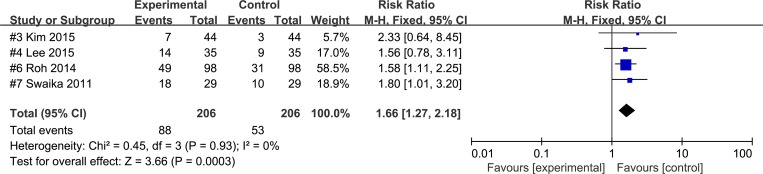
Forest plot for studies comparing the effect of palonosetron and to that of ramosetron on overall PONV when the administration time was at the end of surgery. The figure depicts individual trials as filled squares with relative size of sample size and solid line as the 95% confidence interval of the difference. The diamond shape indicates the pooled estimate and uncertainty for the combined effect.

In several studies in which the 5-HT_3_ antagonist was administered during the early phase of surgery, the effects of palonosetron were compared to those of ramosetron. The combined results of these studies showed that overall PON [14–16,19] (RR 0.66; 95% CI 0.45 to 0.96; P_chi_^2^ = 0.66; I^2^ = 0, NNTB 11.6; 95% CI, NNTB 5.8 to 972.6) and overall POV [14–16,19] (RR 0.46; 95% CI 0.27 to 0.80; P_chi_^2^ = 0.36; I^2^ = 6%, NNTB 9.1; 95% CI, NNTB 5.4 to 28.1) occurred in more ramosetron recipients than palonosetron recipients. However, the combined analysis of the studies in which the drugs were administered at the end of the surgery indicated that overall PON [18,20] (RR 1.43; 95% CI 1.02 to 2.01; P_chi_^2^ = 0.45; I^2^ = 0%, NNTH 8.3; 95% CI, NNTH 4.3 to 133.3) and overall PONV[17,18,20,21](RR 1.66; 95% CI 1.27 to 2.18; P_chi_^2^ = 0.93; I^2^ = 0%, NNTH 5.9; 95% CI, NNTH 3.8 to 12.5) showed higher in palonosetron recipients than ramosetron recipients (Figs 3 and 4).

### Quality of the evidence

Three outcomes (PON, POV and PONV) by three time phases (early, late and overall) with safety analysis (headache and dizziness) in this systematic review were evaluated using the GRADE system. The evidence quality for each outcome was low or very low (Table 5). The quality of pooled analysis for early PON, late PON, overall POV, early PONV and overall PONV showed very low. Otherwise, the quality of pooled analysis for overall PON, early POV, late POV, late PONV, headache, dizziness and all subgroup analysis showed low. The quality of overall PON, POV which antiemetics were administered during early phase of surgery and overall POV and PONV which antiemetics were administered during late phase of surgery showed low.This finding may lower the confidence in any recommendations.

**Table 5:** The GRADE evidence quality for each outcome.

Quality assessment							№ of patients		Effect		Quality	Importance
Outcomes	№ of studies	Risk of bias	Inconsistency	Indirectness	Imprecision	Other considerations	Palonosetron	Ramosetron	Relative (95% CI)	Absolute (95% CI)		
Early PON	6	serious	serious	not serious	not serious	none	81/304 (26.6%)	80/305 (26.2%)	**RR 0.92** (0.54 to 1.58)	**21 fewer per 1,000** (from 121 fewer to 152 more)	⨁◯◯◯VERY LOW	IMPORTANT
Late PON	5	serious	serious	not serious	not serious	none	55/274 (20.1%)	60/275 (21.8%)	**RR 0.87** (0.48 to 1.57)	**28 fewer per 1,000** (from 113 fewer to 124 more)	⨁◯◯◯VERY LOW	IMPORTANT
Overall PON	3	not serious	serious	not serious	not serious	none	108/184 (58.7%)	107/186 (57.5%)	**RR 0.92** (0.54 to 1.58)	**190 more per 1,000** (from 253 fewer to 1,000 more)	⨁⨁◯◯LOW	IMPORTANT
Early POV	6	serious	not serious	not serious	not serious	none	24/297 (8.1%)	32/296 (10.8%)	**RR 0.75** (0.46 to 1.23)	**31 fewer per 1,000** (from 23 more to 64 fewer)	⨁⨁◯◯LOW	IMPORTANT
Late POV	4	serious	not serious	not serious	not serious	none	16/238 (6.7%)	25/237 (10.5%)	**RR 0.66** (0.39 to 1.14)	**37 fewer per 1,000** (from 12 more to 65 fewer)	⨁⨁◯◯LOW	IMPORTANT
Overall POV	4	serious	serious	not serious	not serious	none	37/239 (15.5%)	57/240 (23.8%)	**RR 0.66** (0.42 to 1.03)	**78 fewer per 1,000** (from 199 fewer to 420 more)	⨁◯◯◯VERY LOW	IMPORTANT
Early PONV	5	serious	serious	not serious	not serious	none	90/277 (32.5%)	79/276 (28.6%)	**RR 1.07** (0.60 to 1.92)	**20 more per 1,000** (from 114 fewer to 263 more)	⨁◯◯◯VERY LOW	IMPORTANT
Late PONV	4	serious	not serious	not serious	not serious	none	79/247 (32.0%)	82/246 (33.3%)	**RR 0.92** (0.72 to 1.19)	**27 fewer per 1,000** (from 63 more to 93 fewer)	⨁⨁◯◯LOW	IMPORTANT
Overall PONV	4	serious	serious	not serious	not serious	none	132/212 (62.3%)	103/212 (48.6%)	**RR 1.23** (0.82 to 1.85)	**131 fewer per 1,000** (from 5 more to 233 fewer)	⨁◯◯◯VERY LOW	IMPORTANT
Headache	5	serious	not serious	not serious	not serious	none	31/283 (11.0%)	29/284 (10.2%)	**RR 1.06** (0.66 to 1.70)	**6 more per 1,000** (from 35 fewer to 71 more)	⨁⨁◯◯LOW	NOT IMPORTANT
Dizziness	5	serious	not serious	not serious	not serious	none	27/283 (9.5%)	31/284 (10.9%)	**RR 0.91** (0.55 to 1.49)	**10 fewer per 1,000** (from 49 fewer to 53 more)	⨁⨁◯◯LOW	NOT IMPORTANT
Early administration; Overall PON	4	serious	not serious	not serious	serious	none	28/171 (16.4%)	43/172 (25.0%)	**RR 0.66** (0.45 to 0.96)	**85 fewer per 1,000** (from 10 fewer to 138 fewer)	⨁⨁◯◯LOW	IMPORTANT
Early administration; Overall POV	4	serious	not serious	not serious	serious	none	16/171 (9.4%)	35/172 (20.3%)	**RR 0.46** (0.27 to 0.80)	**110 fewer per 1,000** (from 41 fewer to 149 fewer)	⨁⨁◯◯LOW	IMPORTANT
Late administration; Overall POV	2	serious	not serious	not serious	serious	none	53/133 (39.8%)	37/133 (27.8%)	**RR 1.43** (1.02 to 2.01)	**120 more per 1,000** (from 6 more to 281 more)	⨁⨁◯◯LOW	IMPORTANT
Late administration; Overall PONV.	4	serious	not serious	not serious	serious	none	88/206 (42.7%)	53/206 (25.7%)	**RR 1.66** (1.27 to 2.18)	**170 more per 1,000** (from 69 more to 304 more)	⨁⨁◯◯LOW	IMPORTANT

**CI,** Confidence interval; **RR,** Risk ratio

## Discussion

The results of the current meta-analysis suggest that there is no evidence of difference between the effectiveness of palonosetron and ramosetron in preventing PON, POV, and PONV. When the administration time for the 5-HT_3_ receptor antagonist was during the early phase of the operation, palonosetron was more effective than ramosetron. However, when the administration time was at the end of surgery, ramosetron was more effective than palonosetron. No evidence of differences in headaches or dizziness was found between palonosetron recipients and ramosetron recipients.

The area postrema, or vomiting center, controls and coordinates nausea and vomiting and is located in the lateral reticular formation of the medulla. This center receives various inputs from peripheral pain receptors, the nucleus solitarius, the vestibular system, the cerebral cortex, the chemoreceptor trigger zone, and receptors in the gastrointestinal tract [21]. Serotonin receptor antagonists bind to 5-HT_3_ receptors competitively and selectively in the chemoreceptor trigger zone of the central nervous system and in the gastrointestinal tract, and they are consequently involved in the inhibition of the emetic symptoms [22]. Several serotonin receptor antagonists have been proven to be more effective than traditional antiemetics, including droperidol, metoclopramide, and alizapride, at lowering the incidence of PONV [23]. In the current meta-analysis, two commercially available 5-HT_3_ antagonists, ramosetron and palonosetron, were compared for their effectiveness in preventing PONV. A number of studies comparing the efficacy of these antagonists have produced contradictory results.

The strength of our meta-analysis is that each dose of ramosetron and palonosetron administrated was the same in all of the included studies. Several studies on the effective doses of ramosetron and palonosetron for PONV prophylaxis have been published [24–27]. In studies by Caniotti [24] and Kovac [26], 0.075 mg of palonosetron effectively reduced PONV; this is the same palonosetron dose in our studies. In the study by Lee [27], the effective dose of ramosetron for prophylaxis of PONV in high-risk patients was 0.6 mg, which was higher than the dose in our studies (0.3 mg). However, in the meta-analysis, 0.6 mg of ramosetron showed no greater benefits than 0.3 mg [25]. Also, significantly fewer instances of PONV occurred in the group that received 0.3 mg of ramosetron than in the placebo group [25].

There was considerable heterogeneity in the result of early, late and overall PON. However, there was no heterogeneity in the result of early, late and overall POV. This may be because compared with vomiting, judgments of feeling nausea are subjective, thus led to cause a large variation. Considerable heterogeneity was also found in overall PONV. After performing thorough review, we found out that three studies[14,15,19] among total seven included studies[14,15,17–21] which administered the 5-HT_3_ receptor antagonist during the early phase of the operation showed better efficacy in palonosetron for preventing overall PONV compared to ramosetron. Otherwise, the rest four studies[17,18,20,21], which administered the 5-HT_3_ receptor antagonist at the end of the operation showed reverse outcome. Therefore, we performed the subgroup analysis through administration times. After subgroup analysis, considerable heterogeneity has been resolved.

A subgroup analysis was performed to compare the effects of ramosetron to those of palonosetron on PONV when the administration time was during the early phase and when it was at the end of the surgery. We found that the time at which the 5-HT_3_ antagonist was administered significantly affected the results. When the administration time for the 5-HT_3_ antagonist was during the early phase of surgery, palonosetron was more effective than ramosetron, though the durations of anesthesia varied from 60 to 169 minutes in our meta-analysis. However, when the administration time was at the end of surgery, ramosetron was more effective than palonosetron. This result strengthens Tong’s recommendation for the management of PONV: If ramosetron is used, it is better to administer it at the end of surgery; if palonosetron is used, it is better to administer it at the beginning [28].

Our study had several limitations. First, fewer than 10 studies were included; this may have caused a high error rate. To lower the error rate, all statistical results with substantial heterogeneity were analyzed by using a t-test (Hartung-Knapp-Sidik-Jonkman method) instead of Z-test [29]. Second, only published studies were included in our meta-analysis. Third, considerable heterogeneity has been shown in the results. However, subgroup yielded stable and robust findings. Last, the present study could not improve the low quality of the evidence, which may lower the confidence in any recommendation. Although the subgroup analyses may not apply to the whole studies, this meta-analysis represented the best available method of synthesizing the current evidence. Also, despite these limitations, the present meta-analysis is the first systematic review in which rigorous methodology was applied to compare the efficacy of palonosetron to that of ramosetron in preventing PONV.

## Conclusions

In summary, the prophylactic administration of ramosetron and that of palonosetron showed no evidence of difference in the incidence of overall PON, POV, and PONV. However, sugbroup analysis indicated that palonosetron was more effective than ramosetron when administration time for the 5-HT_3_ antagonist was during the early phase of the operation, and ramosetron was more effective.when the administration time was at the end of surgery. However, due to its small number of included studies and the low quality of the evidence, further randomized controlled trials with profound, well-designed and large scale would be needed.

## Supporting Information

S1 Checklist PRISMA checklist. This is PRISMA 2009 checklist.
(DOC)

S1 Appendix Appendix. This appendix contains the search strategy which combined free text and medical subject heading terms.
(DOCX)
