# Supplementary material for: Palonosetron and Ramosetron Compared for Effectiveness in Preventing Postoperative Nausea and Vomiting: A Systematic Review and Meta-Analysis
Source: PLoS One. 2016 Dec 19;11(12):e0168509. doi: 10.1371/journal.pone.0168509 (PMC5167547; doi:10.1371/journal.pone.0168509)
Supplement: S1 Appendix — This appendix contains the search strategy which combined free text and medical subject heading terms. (DOCX) [file pone.0168509.s002.docx]

**Search terms for MEDLINE**

1. randomized controlled trial.pt
2. randomized controlled trial$.mp
3. controlled clinical trial.pt
4. controlled clinical trial$.mp
5. random allocation.mp
6. exp double-blind method/
7. double-blind.mp
8. exp single-blind method/
9. single-blind.mp
10. or/1-9
11. clinical trial.pt
12. clinical trial$.mp
13. exp clinical trial/
14. (clin$ adj25 trial$).mp
15. ((singl$ or doubl$ or tripl$ or trebl$) adj25 (blind$ or mask$)).mp
16. random$.mp
17. exp research design/
18. research design.mp
19. or/11-18
20. 10 or 19
21. Case report.tw.
22. Letter.pt.
23. Historical article.pt.
24. Review.pt.
25. or/21-24
26. 20 not 25
27. Palonosetron.mp.
28. Aloxi
29. Or/27-28
30. Ramosetron.mp.
31. Ibset.mp.
32. Iribo.mp.
33. Nozia.mp.
34. Nasea.mp.
35. Or/30-34
36. 29 and 35
37. 26 and 36

**Search terms for Embase**

1. randomi?ed controlled trial$.mp.
2. 'controlled clinical trial (topic)'/exp
3. controlled AND clinical AND trials
4. controlled clinical trial$.mp.
5. 'randomization'/exp
6. 'random allocation'/exp
7. random allocation.mp.
8. double-blind.mp.
9. single-blind.mp.
10. #1 OR #2 OR #3 OR #4 OR #5 OR #6 OR #7 OR #8 OR #9
11. 'clinical trial (topic)'/exp
12. clinical AND trial$.mp.
13. random$.mp.
14. rct
15. #11 OR #12 OR #13 OR #14
16. #10 OR #15
17. 'case study'/exp
18. 'case report'/exp
19. 'abstract report'/exp
20. 'letter'/exp
21. #17 OR #18 OR #19 OR #20
22. #16 NOT #21
23. Palonosetron.mp.
24. Aloxi
25. Or/27-28
26. Ramosetron.mp.
27. Ibset.mp.
28. Iribo.mp.
29. Nozia.mp.
30. Nasea.mp.
31. Or/30-34
32. 29 and 35
33. 26 and 36

**Search terms for CENTRAL**

1. (Palonosetron or Aloxi)
2. (ramosetron or Ibset or Iribo or Nozia or Nasea)
3. 1 and 2

**Search terms for Web of Science**

1. TS =(Palonosetron or Aloxi)
2. **TS=**(ramosetron or Ibset or Iribo or Nozia or Nasea)

**Search terms for Google Scholar**

Two separate searches were run using the term (Palonosetron or Aloxi) and (ramosetron or Ibset or Iribo or Nozia or Nasea)

**Search term for Koreamed**

Two separate searches were run using the term (Palonosetron or Aloxi or팔로노세트론 or 알록시) and (ramosetron or Ibset or Iribo or Nozia or Nasea or 라모세트론 or 나세아 or 나제아)
